# Concentrating Emergency Rooms: Penny‐Wise and Pound‐Foolish? An Empirical Research on Scale Economies and Chain Economies in Emergency Rooms in Dutch Hospitals

**DOI:** 10.1002/hec.3409

**Published:** 2016-09-30

**Authors:** Jos L. T. Blank, Bart L. van Hulst, Vivian G. Valdmanis

**Affiliations:** ^1^ Delft University of Technology Delft The Netherlands; ^2^ Erasmus University Rotterdam The Netherlands; ^3^ Western Michigan University Grand Rapids MI USA

**Keywords:** scale economies, chain economies, cost function, emergency rooms, hospitals

## Abstract

In this paper, we address the issue of whether it is economically advantageous to concentrate emergency rooms (ERs) in large hospitals. Besides identifying economies of scale of ERs, we also focus on chain economies. The latter term refers to the effects on a hospital's costs of ER patients who also need follow‐up inpatient or outpatient hospital care. We show that, for each service examined, product‐specific economies of scale prevail indicating that it would be beneficial for hospitals to increase ER services. However, this seems to be inconsistent with the overall diseconomies of scale for the hospital as a whole. This intuitively contradictory result is indicated as the *economies of scale paradox*. This scale paradox also explains why, in general, hospitals are too large. There are internal (departmental) pressures to expand certain services, such as ER, in order to benefit from the product‐specific economies of scale. However, the financial burden of this expansion is borne by the hospital as a whole. The policy implications of the results are that concentrating ERs seems to be advantageous from a product‐specific perspective, but is far less advantageous from the hospital perspective. © 2016 The Authors. *Health Economics* Published by John Wiley & Sons, Ltd.

## 1 Introduction

The recent financial and economic crisis poses major challenges for European countries. They are required to get their national budget in line with strict European Union (EU) budget rules. To meet these challenges, public spending has had to be curbed. The Dutch government has formed multidisciplinary task forces to gain insight into possible savings on public spending. The Task Force on Curative Care (i.e., medical treatment) has researched how to achieve possible savings within the healthcare sector. The target for the task force was to identify € 6.35 billion of potential savings within the medical care 1The medical care sector is considered as hospitals, physicians and other services for direct treatment and does not include any type of long‐term care and public health services such as surveillance, immunization programs, among others. sector (mainly hospitals), equaling 20% of the premium‐financed expenditures in the healthcare sector. These potential savings must be achieved within the policy framework of maintaining accessibility, quality and affordability of healthcare. This is especially challenging as medical care is one of the largest and fastest growing areas of public expenditure. With a real cost growth of 4.5% per year, medical care is an important component of all public spending.

One of the concrete sources of savings that the task force (Task force Curatieve zorg, 2010) identifies is the number of emergency rooms (ER), because there is a significant ER overcapacity. Because every Dutch hospital has an ER, a policy suggestion has been put forward to limit the availability of ERs to one or two per region. This change is designed to result in the better use of ER capacity. The reduction would increase transportation costs (social costs borne by the public, Bernet et al. (2011)) as the average travel time would increase. This can vary strongly by region.

Since 2012, two reimbursement schedules apply for patients visiting an ER: a fixed reimbursement for a medical consultation as well as a fixed reimbursement for any additional activities. The reimbursement for an admission to the ER is a small amount (about € 300) and only covers the expenses of a first diagnosis and stabilizing the patient. Any further treatment is being compensated separately according an extra DBC (Diagnosis Treatment Combination, akin to Diagnostic Related Groups in the U.S. Medicare program) and are fixed; there is no relation with the actual costs. The task force acknowledges that the proposed closure of an ER is unattractive, from a hospital's perspective, as the ER is sometimes the entry point to the hospital for inpatient treatment. What makes the task force's recommendation unpalatable for some hospitals is that they do not necessarily have to close their ER; they only lose funding for the availability of an ER. This would, however, mean that hospitals without a funded ER are at a competitive disadvantage to hospitals as compared with a hospital with a funded ER. The task force does note that these hospitals will have to reconsider their strategic position either by specializing, merging or closing.

Not only has the task force focused on the economics and costs of medical treatment, the Council for Public Health and Health Care (RVZ) has also investigated the healthcare sector. In an advisory report to the Minister of Healthcare, the RVZ has provided an overview of the future of the Dutch hospital landscape (RVZ, 2011a, 2011b) as well as advising concentrating ERs.

Given the discussion above, Dutch policy makers have to decide, first whether to concentrate ERs, and second, how to implement a concentration. However, there is no empirical evidence that economies of scale exist or that they can only be realized in large hospitals. One might even suggest the option of stand‐alone urgent care centers such as those operating in the United States. However, as we noted above, there is no extensive literature on this matter; we, therefore, investigate the existence of economies of scale and chain economies of ER services. Unlike the typical studies of economies of scale and scope, wherein scope is gauged by comparing the costs of producing two independent services (or more) in one firm versus the costs of producing each service in separate firms, chain economies refer to the cost effects of joint production of logical sequential services. The key element of chain economies is that there is a natural of logical dependency between services, that is, in this paper a visit to the ER might result in an admission to the (same) hospital. To summarize: the purpose of this paper is thus to investigate the relationship between cost and the specific services provided in each of the Dutch hospitals in our sample. To do this we answer the following questions:

- Are there economies of scale and/or diseconomies of scale for ERs?
- Is there an optimum scale for ERs?
- What are the effects of the chain economies required for the provision of total hospital care?
- Is it economically advantageous to concentrate ERs in large hospitals?

In order to answer these questions, our research consists of a brief review of the international literature on economies of scale and chain economies in ERs and an empirical analysis of the cost structure of total hospital service where the ER is a part of these services. The subjects of the quality delivered and the concentration and accessibility of ERs are not accounted for. This omission is because of incomplete data on quality and must therefore be addressed in future research.

The paper is organized as follows. In Section 2, we discuss the literature with respect to the scale and scope effects on ERs. In Section 3, we apply a theoretical model, followed by a formal mathematical representation. The details of an empirical application of the model to the Dutch hospital industry are provided in Section 4. The results of the econometric analysis followed by a summary and discussion of these results, in a policy framework, are given in Sections 5 and 6, respectively.

## 2 Literature

The aim of the literature review is to examine what is known about the scale and scope effects of ERs and hospitals from a cost perspective. In contrast to the extensive literature on hospital cost structures, there is a limited number of ER cost studies. Based on a literature, search items including cost (function), scale and scope combined with emergency rooms (units, departments) or hospitals, only a very limited number of studies were found. Only six articles contain results regarding the cost of the ER (Grannemann *et al*., 1986; Baraff *et al*., 1991; Williams, 1996; Bamezai *et al*., 2005; Bamezai & Melnick, 2006; Kim *et al*., 2009). These studies are based on data from hospitals in different regions of the United States, and some are over 20 years old. Given that some of these studies are dated and given that many policy and procedural changes have occurred in hospital care that have led to organizational, technological and reimbursement adjustments, we cite these works as precedence for using a cost function approach. For example, changes in reimbursement formulae have led to increases for primary care, reductions in inpatient days and alternative sources of care may have affected the cost structure of the ERs.

Most of the literature related to ER services, particularly in the US, is focused on the inappropriate use of these services. Even with this focus on the inappropriate use of this expensive venue of care, very little is known about their cost structures. Five studies Grannemann et al. (1986); (Baraff *et al*., 1991; Bamezai *et al*., 2005; Bamezai & Melnick, 2006; Kim *et al*., 2009) use regression methods to determine the cost structure of ERs. Using the economic cost structure, the marginal cost of an emergency unit visit can be derived, that is, the cost of an additional visit to the emergency unit. If the marginal costs are less than the average cost per ER visit, then there are scale economies. As long as marginal costs are less than average costs, additional visits lead to decreasing average costs per ER visit.

Bamezai et al. (2005) demonstrate that most of an ER's costs are comprised of labor costs (85%), but exclude any of the costs of total care such as diagnostic testing (laboratory or radiology services) or hospital admissions. This exclusion limits the value of this approach in ascertaining true ER costs and scale effects. On the basis of a regression analysis, the authors conclude that there is no evidence for economies of scale. Bamezai and Melnick (2006) reach the same conclusion, which is unsurprising as the study is an actualization of the Bamezai et al. (2005) study and uses the same data set.

Grannemann et al. (1986) provide evidence that economies of scale prevail: the marginal costs in this study are about 60% of the average cost at the level of 21,000 ER visits. The scale effects decrease with the number of ER visits. In addition, these authors show that there are diseconomies of scope between the ER and hospital admissions. This implies that hospitals with a large number of admissions will have a relatively higher cost per ER visit. This may be a result of the fact that larger hospitals treat more complex cases in the ER. Another reason for the higher costs maybe that larger hospitals may have more difficulties in coordinating different activities, which in turn can increase costs.

Williams (1996) uses monthly data from six hospitals to estimate the marginal cost of an additional ER visit. According to these estimates, the marginal cost is about half the average cost. Scale economies are present, thus collaborating the findings of Grannemann et al. (1986).

Kim et al. (2009) investigate the cost structure of trauma centers specifically. Trauma units are identified by levels ranging from, Level I, that provides the highest level of surgical care to trauma patients, to Level IV, that only provides initial evaluation, stabilization, diagnostic capabilities and transfer to a higher level of care. The marginal cost is about half the average cost of Trauma Level I and II hospitals, and about a quarter of Level IV hospitals. The average number of visits per year is around 50,000 for Level I and II and around 9000 for Level IV hospitals. The study shows that economies of scale exist for Trauma Levels I to IV.

Even though there are only a few research examples in the literature on ER cost studies, there appear to be consistent findings with respect to the economies of scale of ERs. Most of these studies rely on data with ERs with 20,000 to 40,000 visits per year. Only in the study by Grannemann et al. (1986) are there indications of economies of scale, but these are eliminated as the number of visits increases or the size of the hospitals (in terms of admissions) increases.

What we add in this study is the establishment of whether economies of scale are present when ERs are regionally concentrated in large hospitals. Concentrating ERs in large hospitals may consequently incur higher costs from the associated diseconomies of scale present throughout the entire hospital. In other words, cost savings can be achieved by enlarging the ERs. However, these savings may disappear because of the higher marginal costs of an admission to a larger hospital. Unlike Grannemann et al. (1986) who focused on economies of scale and scope, we expand and look at a more realistic view of hospital care by examining economies of scale and chain economies which includes the total cost of patients entering the ER and either being discharged, admitted to the hospital or referred to outpatient services. In this way, we can examine if affecting the scale economies of one service would increase, decrease or change in other ways, the economies of scale in other services. Hence, just closing an ER may affect the economies of scale of other hospital services, negating any cost benefit.

## 3 Theoretical Model

The effects of scale and scope on the costs of the Emergency Room can be established by analyzing the cost structure of hospitals. As Grannemann et al. (1986) suggest, we also use the hospital as the unit of analysis for the cost function which presents the (mathematical) relationship between the costs and the size and composition of services, the price of inputs (such as the salaries of nurses) and the medical technology used for ER care. The cost function is defined as:

(1) $$c\left(y,w\right)=\min_{x}\left\{w\cdot x|x\;\epsilon \;P\left(y\right)\right\}$$

With:

*y*: = vector of services;
*x*: = vector of resources;
*w*: = vector of resource prices;
*P*(*y*): = input set belonging to *y*.

By choosing a functional form for c(*y*,*w*), the parameters of the cost function can be estimated. From the estimated cost function, we can derive the following relationships:

- (multiproduct) economies of scale;
- product‐specific economies of scale;
- chain economies.

Economies of scale can be derived by taking the inverse of the cost elasticity with respect to services:

(2) $$v={\left[\sum_{m}\frac{\partial \log c\left(y,w\right)}{\partial \log y_{m}}\right]}^{-1}$$

When the scale elasticity is greater than one, economies of scale exist. The relative change in cost is lower than the relative change in the production of services. If the scale elasticity is less than one, diseconomies of scale prevail. If the scale elasticity equals one, neither economies nor diseconomies of scale exist which is deemed as constant returns to scale.

We can re‐write equation (2) as:

(3) $$v=\frac{c\left(y,w\right)}{\sum_{m}\;y_{m}\frac{\partial c\left(y,w\right)}{\partial y_{m}}}$$

According to (3) the elasticity of scale equals the ratio of total cost and the weighted sum of marginal costs over all distinct services. The weights are determined by the point values of the services.

In addition to multi‐product economies of scale, it is possible to derive the so‐called product‐specific economies of scale for each separate service. The expansion of a particular service lowers average cost for the service. For illustrative purposes, we present the two‐dimensional case, where we derive the product specific economies of scale (or scale elasticity) for two individual services. From this application, we are able to express product‐specific economies as follows:

(4) $$v\left(y_{2}|y_{1}\right)=\frac{\frac{c\left(y_{1},y_{2}\right)-c\left(y_{1},0\right)}{y_{2}}}{\frac{\partial c\left(y_{1},y_{2}\right)}{\partial y_{2}}}$$

The numerator reflects the average cost for producing service 2, whereas the denominator reflects the marginal cost of producing one extra unit of y_2_. The interpretation of this product specific scale elasticity is consistent with determining overall scale elasticity.

It is not always possible to establish the cost of zero production of one of the services empirically. As we will see in our application there are no Dutch hospitals without an ER, hence we apply the following alternative for equation (4):

(5) $$v\left(y_{2}|y_{1}\right)=\frac{\frac{c\left(y_{1},y_{2}\right)-c\left(y_{1},y_{2}^{min}\right)}{y_{2}-y_{2}^{min}}}{\frac{\partial c\left(y_{1},y_{2}\right)}{\partial y_{2}}}$$

The zero production in the cost function in (4) is replaced by the lowest number of services $y_{2}^{min}$ in the data set. Because fixed costs as a result of the production of $y_{2}^{min}$ are also included in the expression $c\left(y_{1},y_{2}^{min}\right)$, it is expected that the scale elasticity according to (5) may be an underestimate of (4).

Note that the outcome of (5) not only depends on the level of service 2, but is also conditional to the level of service 1. To gain insight into this relationship, we take the derivative of *v*(*y*_2_|*y*_1_) in equation (5) with respect to *y_1_*. In general, this expression is negative, implying that increasing returns to scale (IRTS) are diminishing with increasing services and finally turning into decreasing returns to scale (DRTS). In empirical applications, taking the derivative of (5) will be relatively complex, particularly when a translog specification is used. In this case, a numerical representation can be applied as an alternative.

In addition to the conditionality of the product‐specific economies of scale, the other key effect to be addressed is the chain economies, which refer to the cost effects of the joint production of two sequential services. This should be distinct from joint production in general, in which the products are being delivered independently. The sequential dependence is the key element here and can be regarded (toward our knowledge) as a new concept. In the ER context, some of the patients arriving at the ER will need to be hospitalized. In this case, an interesting question arises as to whether economies of scale exist with respect to the joint production of both types of services. Les us assume that *y_12_* represents the number of patients admitted via the ER. Further note that this number is a part of the number of ER visits, as well as a part of the number of admissions. In that case—analogous to the definition of product‐specific economies of scale—chain economies are defined as:

(6) $$v_{12}=\frac{\frac{c\left(y_{1},y_{2}\right)-c\left(y_{1}-y_{12},y_{2}-y_{12}\right)}{y_{12}}}{\frac{\partial c\left(y_{1},y_{2}\right)}{\partial y_{1}}+\frac{\partial c\left(y_{1},y_{2}\right)}{\partial y_{2}}}$$

With:

*y_12_*: = number of joint services 1 and 2 (e.g., admissions via the ER).

Equation (6) states that economies of scale with respect to the joint production of service 1 and service 2 equal the ratio of the average incremental cost of producing *y_12_* and the marginal cost of producing an extra unit of *y_1_* and an extra unit of *y_2_*. Note that from a policy point of view, an interesting case occurs when *v_2_* > 1 (economies of scale) and *v_12_* < 1 (diseconomies of scale). From the narrow perspective of emergency visits, expanding services leads to lower average costs, whereas from the chain perspective expanding services leads to higher total average costs.

## 4 The Empirical Model

### 4.1 The Dutch hospital industry

In the Dutch hospital system, there are three types of hospitals: general hospitals, academic hospitals and specialty hospitals. We limit out study to general hospitals because academic hospitals and specialty hospitals differ from general hospitals so much that any findings including these hospitals would suffer from heterogeneity bias. Omitting these specialty and sophisticated hospitals does not appear to affect eventual degrees of freedom because general hospitals comprise 80% of hospital beds and almost 70% of Dutch hospital costs. General hospitals have various facilities for diagnostics, treatment and nursing as well as for the training of physicians and nurses. (They differ from academic centers by not admitting patients who need extremely complex and very expensive treatments).

ERs are medical treatment facilities treating acute care of patients who present themselves without prior appointment. In the Dutch system, hospitals are the sole providers of acute care in ERs. As a result, ERs are always located inside a hospital building. In practice, all hospitals in our sample have an ER. An important feature of ERs is their availability; all ERs (except one) are open 24 hours a day, 7 days a week. Most patients are referred to the ER by a professional, still about one third of the patients are self‐referrals. About 15% of all patients are acute or highly urgent, 40% is urgent, 39% is semi‐urgent and 3% is non‐urgent (for the other 3% the urgency is unknown).

Since 2005, the Dutch hospital industry has a system with product classification. Patients are classified based on their diagnoses and treatments. The product that is derived from this classification is the so‐called DBC (Diagnostic Treatment Combination), similar to the Diagnostic Related Groups (DRGs) applied in the U.S. and in other European countries. From an economic perspective there are two types of DBC, the A‐segment for which the price is regulated and set by the government and the B‐segment for which prices are negotiated by hospital and health insurance providers. Hospitals negotiate with health insurers on price, volume and quality.

Another feature of the Dutch hospital sector is that hospitals cannot select their patients. Patients are referred to a hospital by general practitioners (GPs). In some cases, the GP can also refer a patient to an ER. Typically, however, patients arrive for treatment in an ER as a self‐referral, based on location and the availability of appropriate specialties. Hospitals are obliged to treat any patient presented to them, provided that they possess the medical capacity required for the treatment. In practice, hospitals can attract patients by supplying particular specialties or a high quality of care. This latter point is salient to the arguments put forth by insurance companies that higher quality is directly related to higher levels of volume, as part of the argument for consolidating ERs into larger units in larger hospitals.

In modeling the costs of hospitals, we have to pay special attention to the cost of physicians. The reason for this is that in the Dutch system there are two types of physician in the hospital, those who are employed by the hospital and those who are self‐employed. In practice, most physicians are self‐employed and liaise with the hospital as entrepreneurs. The costs and funding of these physicians vary between hospitals. One drawback of this arrangement is that data on the costs of the self‐employed medical specialist are not available. In an empirical application, we must therefore exclude the costs of (all) physicians.

### 4.2 Model implications and data availability

In our cost function model, the dependent variable is the total costs of the hospital. Because the costs of medical specialists are separated from hospital costs, these costs are excluded from the analysis. Because hospitals are not allowed to make any profits, but are forced to meet the available budgets (A‐segment) or compete based on service prices (B‐segment), we assume cost minimizing behavior.

Because patients are free to choose their hospital, the services delivered are exogenous for hospitals. For hospital outputs, we use the familiar service delivery of hospitals measured by the number of admissions and outpatients (see for example Blank & van Hulst (2009)), the volume of research activities and other services and visits to the ER. The volume of research activities and other services is measured by the revenues that are not generated by treating patients. Only including admissions, outpatients and ER visits as output does not account for the heterogeneity of the production of hospitals. We therefore apply a hedonic‐index (Lancaster, 1966) that accounts for the characteristics of the hospital. The hedonic‐index contains the following elements: relative size of the surgery and orthopedic departments (measured by the number of physicians), expected length of stay (based on the mix of specialties available in the hospital), number of intensive care (IC) beds, the presence of a psychiatric ward, the presence of neurosurgery and the presence of cardiothoracic surgery. The hedonic‐index is a straightforward tool that accounts for case‐mix differences among hospitals. The admissions included in the cost function are weighted by the hedonic index and credits hospitals with a more severe case‐mix in accounting for cost differentials.

Resources include four categories of labor, material supplies and capital. The sum of the costs of these resources adds up to the total costs. Furthermore, data on prices for these resources are needed for successful estimation of the cost function and the cost shares.

The following four categories of labor are distinguished: management and administrative personnel, nursing personnel, paramedical personnel (e.g., lab technicians, psychologist) and auxiliary personnel (e.g., hotel personnel, security, cleaning). The implicit assumption of excluding medical specialists is that there are no substitution possibilities between physicians and other personnel, because a physician practice is a protected profession and conduct procedures that other personnel cannot. For all hospitals, data are available on the costs and the quantity for each personnel category. The price for each personnel category is derived from its unit value: the quotient of cost and number of FTEs. Because unit values it selves are partly endogenous we establish labor prices by regressing unit values on (health) region and year dummies. The predicted values are then used as proxies for exogenous price differentials and considered as the market prices for labor.

Material supplies include medical supplies, food and heating. Because there is no natural unit of measurement for material supplies, a circumventing construction was used. For materials, we use an index. For the first year in the dataset the price of materials is set at one, we assume there is no price variation for materials between regions. Because the Netherlands is a small country, this a reasonable assumption. In the following years, the price of material supplies is adjusted by the consumer price index for the Netherlands as calculated by Statistics Netherlands.

Capital consists of assets such as buildings and medical equipment. There are data available on the costs of capital and indicators that represent the volume of capital. The price of capital is derived from the cost of capital divided by the volume of capital. The latter is a volume index based on the weighted aggregation of the number of beds, intensive care beds, radiotherapists (proxy for the number of linear accelerators and cobalt machines) and operating theaters. The weights for the volume index are derived from a regression of capital costs on the variables that comprise the volume index.

### 4.3 Descriptive statistics of the data

All hospitals are required to submit annual reports containing information on costs, production and specific characteristics of the hospital. In addition to the annual reports, there is a yearly survey containing information on specific resources and some other characteristics of the hospitals. Data from these annual reports are freely available; the additional data from the survey were obtained from the NVZ (Dutch General Hospital Association). Although the data from both sources are quite extensive, they do not contain any information on visits to ERs. Therefore, we used an additional survey among General Hospitals to obtain information on visits to the ERs. More than 80% of the hospitals responded to the survey.

The data from the three sources are combined resulting in a dataset suitable for our analysis. The time range of the three datasets, however, differs. Information on costs and production ranges from 2003 to 2011, while the data on emergency visits only ranges from 2007 to 2012. Furthermore, after checking the data for unreliable observations or outliers, some of the observations had to be removed, resulting in unequal number of observations for each year. The dataset on costs and production contains 682 observations. The cross section of costs and production and the data on ER visits contains 249 observations.

Table 1 contains the descriptive statistics for the variables used in the cost function. The descriptive statistics are from 2011, the most recent year that includes all items in the dataset.

**Table 1:** Descriptive statistics, Dutch General Hospitals 2011 (*N* = 67)

	Mean	Std. dev.	Min.	Max.
Admissions	44,767	20,854	16,768	106,549
Outpatients	76,347	29,048	31,247	159,810
Other revenues	14,896	10,079	1,982	57,959
ER visits^a^	24,115	12,771	7,943	74,532
Surgery and orthopedics (%)	11.7	2.4	3.8	20.0
Psychiatric beds/ 1000 admissions	0.27	0.40	—	1.54
IC‐beds per 1000 admissions	0.23	0.09	—	0.50
Expected length of stay	3.3	0.3	2.5	4.3
Neurosurgery (%)	0.8	1.9	—	12.7
Cardiothoracic surgery (%)	0.3	0.9	—	4.2
Total costs (× 1 million €)	147.8	78.2	52.2	364.3
*Cost shares*				
Management/ administration	0.10	0.02	0.03	0.16
Nurses	0.34	0.04	0.27	0.46
Paramedics	0.03	0.02	0.00	0.08
Auxiliary personnel	0.09	0.02	0.02	0.16
Material	0.34	0.03	0.22	0.40
Capital	0.09	0.02	0.04	0.16

a *N* = 55.

In 2011, hospitals had, on average, 76.3 thousand outpatients, 44.8 thousand admissions and 24.1 thousand visits to the ER. Compared with 2003 the number of outpatients has grown by 30%, while the number of admissions has grown by 66%. The average costs for hospitals in 2011 are 147.8 million Euros; in nominal terms, this is an increase of 57% since 2003. Most of the resources are allocated to nurses and materials, which both have a cost share of 34%.

An ER visit may be followed by an inpatient admission or outpatient care. In order to take into account this combination of products that are provided sequentially, we use the notion of chain economies. After an ER visit, there is a variety of follow‐up scenarios. Table 2 gives the descriptive statistics for follow‐up scenarios.

**Table 2:** Descriptive statistics, share of patients' treatment disposition following an ER visit

	Mean	Std. dev.	Min.	Max.
No follow‐up	0.35	0.19	0.01	0.69
Outpatient	0.27	0.17	0.03	0.53
Admission	0.32	0.08	0.14	0.46
Admission IC/ stroke unit/ CCU	0.03	0.03	—	0.15
Admission other hospital	0.01	0.01	—	0.02
Other	0.03	0.03	—	0.10

## 5 Estimation and Evaluation

### 5.1 Specification

In previous research set in the Dutch context, a cost minimizing model was employed wherein services, resource prices and capital inputs are treated as exogenous variables (see e.g., Blank & van Hulst, 2009). In line with this earlier work, we estimate a direct cost function model made up by a cost function and a number of cost share equations (see Section 2). In order to simplify the interpretation of the estimated parameters, all variables in the analysis are standardized at their arithmetical means. The first‐order parameter estimates then represent the cost elasticity with respect to the corresponding service or resource price for the ‘average’ hospital.

The models are estimated using multivariate regression techniques using various equations with a joint density, which we assume to be normally distributed. Because disturbances are likely to be cross‐equation correlated, a minimum distance estimator is used. Because the shares add up to one causing the variance–covariance matrix of the error terms to be singular, one share equation in the direct cost function model is eliminated.

Because of the large number of parameters to be estimated with respect to the number of observations, we apply a two‐stage procedure. As we have explained in Section 4.3, only a subsample of the data includes data on ERs. If we omit the observations with missing values on the ER related variables, a loss of degrees of freedom occurs affecting the efficiency of the parameter estimates. In the first stage, we estimate the model without the ER related variables on the complete dataset. In the second step, we re‐estimate the model on the subsample (this includes all the variables), fixing all the parameters of the terms corresponding to the resource prices to their estimated values. By doing this, we implicitly assume that resource prices and services are uncorrelated. We also validated this assumption by following the same procedure on the services variables without the emergency services. We found that the parameters did not significantly change by applying this assumption.

### 5.2 Results

In Table 3, we present the estimates of the first and second stage of the model.

**Table 3:** Parameter estimates first stage and second stage

Parameter	Stage 1			Stage 2		
	Estimate	St. error	t‐statistic	Estimate	St. error	t‐statistic
Constant	0.203	0.015	13.594	0.191	0.008	23.463
2004	−0.035	0.010	−3.501			
2005	−0.056	0.010	−5.451			
2006	−0.081	0.011	−7.389			
2007	−0.095	0.013	−7.249			
2008	−0.123	0.014	−8.609			
2009	−0.138	0.015	−9.115			
2010	−0.146	0.016	−9.237			
2011	−0.172	0.017	−10.184			
Admissions	0.626	0.021	30.330	0.577	0.021	27.465
Outpatients	0.353	0.021	16.455	0.337	0.031	10.995
Other revenues	0.089	0.008	10.896	0.102	0.011	9.016
ER visit				0.034	0.018	1.834
Admissions × Admissions	−0.085	0.060	−1.403	0.020	0.035	0.564
Admissions × Outpatients	0.139	0.060	2.295			
Admissions × Other revenues	0.019	0.023	0.794			
Outpatients × Outpatients	−0.128	0.086	−1.487	0.045	0.070	0.643
Outpatients × Other revenues	−0.036	0.025	−1.456			
Other revenues × Other revenues	0.010	0.009	1.188	0.022	0.010	2.182
ER visit × ER visit				−0.038	0.035	−1.081
Price man. & adm.^a^	0.116	0.003	36.426			
Price nursing personnel	0.338	0.005	63.192			
Price paramedical personnel	0.053	0.002	22.808			
Price auxiliary personnel	0.093	0.004	25.585			
Price material supplies	0.302	0.006	47.997			
Price capital	0.098	0.002	64.879			
Price man. & adm. × price man. & adm.	0.016	0.015	1.094			
Price man. & adm. × price nursing personnel	0.094	0.018	5.163			
Price man. & adm. × price medical personnel	0.026	0.007	3.918			
Price man. & adm. × Price auxiliary personnel	−0.002	0.012	−0.183			
Price man. & adm. × price material supplies	−0.127	0.018	−6.899			
Price man. & adm. × price capital	−0.007	0.003	−2.476			
Price nursing personnel × price nursing personnel	−0.117	0.046	−2.554			
Price nursing personnel × price medical personnel	0.011	0.011	0.992			
Price nursing personnel × price auxiliary personnel	−0.041	0.021	−1.916			
Price nursing personnel × price material supplies	0.076	0.039	1.944			
Price nursing personnel × price capital	−0.024	0.004	−5.653			
Price medical personnel × price medical personnel	−0.014	0.006	−2.212			
Price medical personnel × price auxiliary personnel	0.015	0.008	1.876			
Price medical personnel × price material supplies	−0.041	0.012	−3.295			
Price medical personnel × price capital	0.003	0.002	1.315			
Price auxiliary personnel × price auxiliary personnel	−0.008	0.019	−0.432			
Price auxiliary personnel × price material supplies	0.047	0.021	2.287			
Price auxiliary personnel × price capital	−0.010	0.003	−3.034			
Price material supplies × price material supplies	0.063	0.048	1.320			
Price material supplies × price capital	−0.018	0.004	−4.351			
Price capital × price capital	0.056	0.002	32.465			
Admissions × price man. & adm.	−0.001	0.003	−0.193	0.001	0.004	0.190
Admissions × price nursing personnel	−0.022	0.004	−4.983	−0.020	0.006	−3.207
Admissions × price medical personnel	0.011	0.002	4.662	0.004	0.003	1.213
Admissions × price auxiliary personnel	−0.007	0.003	−2.135	−0.009	0.004	−2.113
Admissions × price material supplies	0.028	0.004	6.289	0.029	0.006	4.695
Admissions × capital	−0.009	0.002	−5.012	−0.005	0.003	−1.940
Outpatients × price man. & adm.	0.011	0.003	3.364	0.013	0.006	2.073
Outpatients × price nursing personnel	0.016	0.005	3.142	0.023	0.009	2.547
Outpatients × price medical personnel	−0.001	0.003	−0.350	−0.005	0.005	−0.972
Outpatients × price auxiliary personnel	−0.003	0.004	−0.833	−0.012	0.007	−1.809
Outpatients × price material supplies	−0.038	0.005	−7.419	−0.033	0.009	−3.522
Outpatients × capital	0.015	0.002	7.156	0.014	0.004	3.518
Other rev. × price man. & adm.	−0.002	0.001	−1.366	0.000	0.002	−0.212
Other rev. × price nursing personnel	−0.003	0.002	−1.757	0.000	0.003	−0.155
Other rev. × price medical personnel	0.006	0.001	5.673	0.005	0.002	2.885
Other rev. × price auxiliary personnel	−0.001	0.001	−0.616	−0.003	0.002	−1.468
Other rev. × price material supplies	0.004	0.002	2.320	0.003	0.003	1.030
Other rev. × capital	−0.004	0.001	−5.789	−0.004	0.001	−2.962
ER visit × price man. & adm.				−0.002	0.004	−0.443
ER visit × price nursing personnel				−0.025	0.006	−4.105
ER visit × price medical personnel				0.013	0.003	3.914
ER visit × price auxiliary personnel				0.021	0.005	4.677
ER visit × price material supplies				−0.006	0.006	−0.974
ER visit × capital				−0.001	0.003	−0.494
Trend × price man. & adm.	0.087	0.022	4.028			
Trend × price nursing personnel	0.049	0.034	1.446			
Trend × price medical personnel	0.061	0.015	4.020			
Trend × price auxiliary personnel	0.028	0.023	1.204			
Trend × price material supplies	−0.251	0.045	−5.544			
Trend × price capital	0.027	0.011	2.442			
Admissions × Surgery and orthopedics	−0.163	0.026	−6.192			
Admissions × Psychiatric beds	0.015	0.003	4.781			
Admissions × IC beds	0.033	0.011	3.105			
Admissions × Expected length of stay	0.254	0.063	4.003			
Admissions × Neurosurgery	0.034	0.004	7.957			
Admissions × Cardiothoracic surgery	0.123	0.011	11.578			

a Price man. & adm—price management and administration.

The cost function model presented in Table 3 fits the data well. The statistical evidence regarding the goodness of fit include: a high *R* ^2^, that is, 0.98, more than 70% of the estimated parameters are significant at the 5% level and most of the outcomes are in line with previous results (Blank & van Hulst, 2009; Blank & van Hulst, 2011). The requirements concerning monotonicity and concavity are also mostly met. The monotonicity property tells us that input demand is always positive, which is the case for almost all observations. Only in a few cases was the positive input demand for capital not met. A necessary condition for concavity is the negativity of the ‘particular’ elasticities of substitution. This condition also holds for the ‘average’ hospital and is valid in 93% of the observations. Finally, the condition of negative semi‐definite of the matrix of elasticities of substitution holds for the average hospital and is also valid in 67% of the observations. For most observations, there is only one value slightly greater than zero.

In order to gain more insight into the plausibility of the estimates, we also present the calculated marginal cost. The marginal cost for each of the hospitals in 2011has been calculated. Quartiles of the predicted marginal cost for each service are given in Table 4.

**Table 4:** Estimated marginal costs, 2011 (in €)

	1st Qrt	Median	3rd Qrt
Admissions	1667	1836	1969
Outpatients	528	586	685
Other revenues	0.92	1.13	1.40
ER visits	150	215	335

From the results presented in Table 4, we note that the marginal cost of admissions vary between € 1670 and € 1970. The marginal cost of the ER visits that vary between € 150 and € 335.

The main results of this analysis are presented in Table 5, because it represents the elasticities of scale for the hospital as a whole, the product specific elasticities of scale and the chain elasticities of scale. Table 5 presents the results for the median and both quartiles.

**Table 5:** Estimated overall and product‐specific scale elasticities, 2011

	1st Qrt	Median	3rd Qrt
Total hospital	0.942	0.959	0.979
*Product specific*			
Admissions	1.093	1.147	1.208
Outpatients	1.147	1.197	1.291
Other revenues	1.454	1.570	1.727
ER visits	1.635	2.320	2.982
*Chain*			
ER visits—admissions	1.059	1.060	1.062
ER visits—outpatients	1.103	1.117	1.125

The results presented in Table 5 show that hospitals face diseconomies of scale. The range of the overall scale elasticities varies between 0.942 and 0.979. If we assess the product‐specific economies of scale, a completely different picture emerges. In the case of product specific scale elasticities, we see that there are economies of scale for all specific products (all product specific scale elasticities are greater than one).

However, ER care is often not a single product and is jointly produced with other services, which we dub chain economies. The scale elasticity for this chain varies for Q1–Q3 between 1.059 and 1.062. The chain economies for ER visits—outpatients vary for Q1–Q3 between 1.103 and 1.125. This is substantially lower than the reported product specific scale elasticities.

## 6 Summary and Conclusions

In this paper, we investigate whether it is economically advantageous to concentrate ERs in large hospitals. A cost function model for hospital care, in which ER visits are included as one of the outputs, is applied to answer this question. For the purposes of this paper, both economies of scale as well as economies of chain are analyzed; here, the economies of chain refers to effects of ER patients who need follow‐up care in the hospital. This summary is based on the findings we report showing marginal cost of admissions varies between € 1670 and € 1970 with a marginal cost of the ER visits that varies between € 150 and € 335.

Regarding the economies of scale, our results are clear that economies of scale for ER services are present. It is therefore beneficial for hospitals to increase ER services. Because we find product‐specific economies of scale for ER services for the full range of hospitals, we cannot give a decisive answer about the optimal size of the ER. The conclusion drawn from these results is that the optimal size is larger than the largest ER in our data set, which equals 75,000 visits.

Turning next to the chain economies, when they are taken into account, the results are less indisputable. Both the distinct chain ER visits (ER followed by inpatient treatment and ER followed by outpatient treatment) face economies of scale but to a far lesser degree than the product‐specific scale elasticities suggest. Although the elasticities are still greater than one, one may wonder whether it is still useful to recommend concentration of ERs. In particular when one also takes the transition costs and geographical proximity issue into account. Even though we cannot conclude from our results that the ER inpatient care nexus leads to increased hospital costs, it is clear that chain economies provide a more nuanced picture of cost consequences.

In addition to product‐specific economies of scale for ER visits, we find product‐specific economies of scale for all distinct services, as all estimated product‐specific scale elasticities are greater than one. These results indicate that it would be beneficial for hospitals to increase the size of each service. The overall scale elasticities for Dutch hospitals vary between 0.94 and 0.98 implying that, from an economic point of view, Dutch hospitals are too large. From a policy perspective concentration through mergers or closures is therefore undesirable for Dutch hospitals. So there seems to be a contradiction between product‐specific economies of scale and the overall diseconomies of scale for the hospital as a whole. This contradictory result could be called the *economies of scale paradox*. This scale paradox explains why, in general, hospitals grow to be too large. There are internal (departmental) pressures to expand certain services, such as ER, in order to benefit from the product‐specific economies of scale. However, the burden of this expansion comes at the expense of the hospital as a whole. It may be hypothesized that product specific economies of scale are connected to improvements in occupancy rates (for instance of capital), whereas overall (dis)economies of scale are related to bureaucratic tendencies in large and complex organizations.

From a policy perspective, it does not seem to be wise to implement incentives or measures that lead to concentration of ERs such as limiting ERs to one or two per region. Although there are economies of scale for ERs, the advantages are offset if chain‐economies are neglected. If chain economies are included the advantages of concentrating ERs are much weaker and almost disappear. If we assume that that the concentrated ERs will be situated in large hospitals, any potential economies of scale that arise from this concentration could be eliminated by hospital overall diseconomies of scale. Still there might be some small advantages, but they may not balance out with the transition costs of concentrating and less geographical access to an ER, thereby shifting costs to patients. Whereas concentrating ERs initially seems attractive, our results suggest a far more nuanced view point.

More specificity between the degree of chain economies among services in the hospital would be another topic for future research. For example, certain services such as orthopedics and surgery may be more tightly linked to ER care, whereas other services such as hematology may not be as close. By addressing these specific service chains, research can provide results to address other policy proposals such as downsizing hospitals deemed to be too large or decreasing underutilized capacity in certain services.

In addition to the supply side issues regarding the ER consolidation, demand side issues also need to be taken into account. Geographical access, such as the time to get to appropriate emergency care, also needs consideration. There are some conditions such as heart attacks and the golden hour for stroke care in which time is crucial. If consolidation increases average time, the added costs of the possible associated acuity of a patient's illness would also negate any decrease in costs by consolidation. Therefore, the time incurred by the patient also needs to be more closely determined such as including how time is measured—ambulance time or time from diagnosis to treatment.

Our results are preliminary and while leading to certain conclusions may be premature, alternatives can be devised using examples from other health care systems and expanded study, simply closing ERs could be penny‐wise but pound‐foolish.
